# Letter from the Editor in Chief

**DOI:** 10.19102/icrm.2025.17028

**Published:** 2026-02-15

**Authors:** Devi Nair


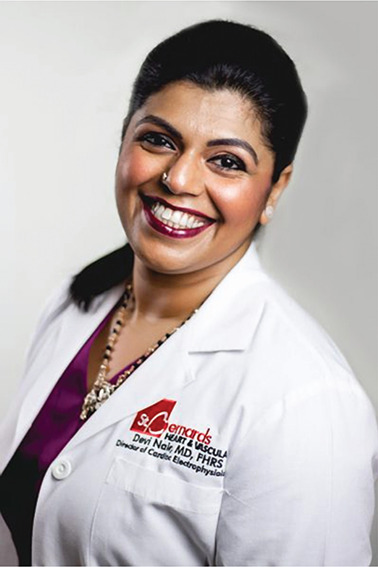


Dear Colleagues,

Welcome to the February 2026 issue of *The Journal of Innovations in Cardiac Rhythm Management*.

February is always an energizing month in electrophysiology, as it coincides with the annual AF Symposium in Boston, a meeting that continues to shape global dialogue around atrial fibrillation management. The 2026 program once again highlighted the evolution of pulsed field ablation platforms, durability discussions, workflow optimization, and the expanding role of adjunctive imaging and mapping technologies. Importantly, the tone of this year’s meeting reflected maturity in the field: there was not only enthusiasm for innovation but also critical appraisal of long-term outcomes, safety signals, and patient selection. These themes resonate strongly with the scholarship presented in this issue.

This month’s manuscripts span the full spectrum of electrophysiology, from mechanistic tracings to complex congenital anatomy and microvascular pathophysiology.

In the work by Al-Shammari et al.,^1^ we are presented with a fascinating case of premature ventricular complex–induced full pre-excitation in Wolff–Parkinson–White syndrome. The report provides invasive electrophysiologic confirmation of a premature ventricular complex rendering the atrioventricular node refractory, allowing exclusive antegrade conduction via the accessory pathway. The case elegantly demonstrates how dynamic conduction changes can clarify mechanism and guide precise ablation, reminding us that careful interval analysis remains foundational, even in the era of advanced mapping.

In this month’s Tracing of the Month, Mondal et al.^2^ dissect an atypical atrioventricular nodal re-entrant rhythm. The case highlights dual atrioventricular nodal physiology, slow–fast pathway interactions, and the interplay between sinus node dysfunction and re-entry. The detailed electrogram analysis reinforces the importance of intracardiac interpretation in distinguishing rhythm from tachycardia, a nuanced but clinically meaningful distinction.

Device therapy is prominently featured in the report by Kesriklioglu et al.,^3^ demonstrating the feasibility of left bundle branch area pacing via a persistent left superior vena cava in a young patient with repaired ventricular septal defect. This case underscores the expanding boundaries of conduction system pacing, even in the presence of complex venous anatomy and prior surgical repair. As physiologic pacing strategies continue to evolve, such reports provide valuable technical insight into adapting tools and technique to challenging substrates.

Weyand et al.^4^ offer a compelling “Images in Cardiac EP” case describing small-vessel disease in a patient with atrial flutter and reduced ejection fraction. The histopathological findings suggest a potential microvascular contributor to tachycardia-induced cardiomyopathy, raising important questions about interindividual susceptibility and reversibility. As rhythm-control strategies improve, understanding underlying myocardial vulnerability becomes increasingly relevant.

Finally, Ozkan et al.^5^ report a case of wide QRS complex tachycardia with alternating ventriculoatrial intervals, presenting a masterclass in interval-based reasoning. Through meticulous electrophysiologic analysis, the mechanism is clarified as an accessory pathway–mediated tachycardia. The case reminds us that, even in complex presentations, careful attention to sequence and timing remains our most powerful diagnostic tool.

Taken together, this issue reflects the diversity of modern electrophysiology: mechanistic insight, procedural innovation, congenital complexity, and microvascular pathology. The discussions unfolding at the AF Symposium, around durability, workflow refinement, physiologic pacing, and substrate characterization, mirror the intellectual rigor reflected in these pages.

As our field continues to evolve rapidly, it is the integration of careful observation, physiologic understanding, and technical innovation that drives meaningful progress. I thank our authors and reviewers for contributing to this ongoing dialogue, and I thank you our readers for your continued engagement.

Warm regards,


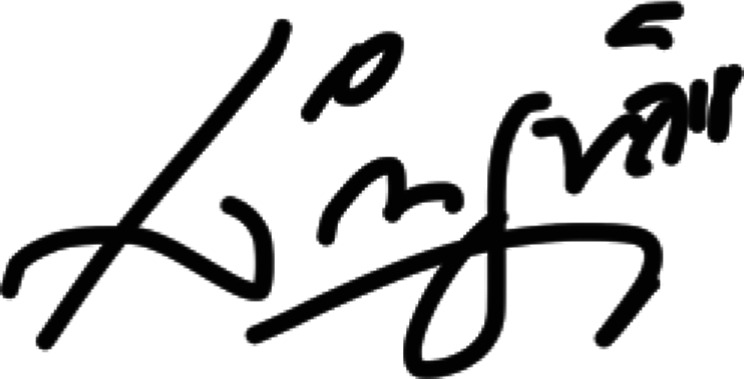


Dr. Devi Nair, md, facc, fhrs

Editor-in-Chief

*The Journal of Innovations in Cardiac Rhythm Management*

Director of the Cardiac Electrophysiology & Research,

St. Bernard’s Heart & Vascular Center, Jonesboro, AR, USA

White River Medical Center, Batesville, AR, USA

President/CEO, Arrhythmia Research Group

Clinical Adjunct Professor, University of Arkansas for Medical Sciences

Governor, Arkansas Chapter of the American College of Cardiology

drdgnair@gmail.com
